# Role of WAVE3 as an actin binding protein in the pathology of triple negative breast cancer

**DOI:** 10.1002/cm.21898

**Published:** 2024-07-18

**Authors:** Kruyanshi Master, Lamyae El Khalki, Mekki Bayachou, Khalid Sossey‐Alaoui

**Affiliations:** ^1^ Department of Chemistry Cleveland State University Cleveland Ohio USA; ^2^ MetroHealth System Cleveland Ohio USA; ^3^ Case Western Reserve University Cleveland Ohio USA; ^4^ Case Comprehensive Cancer Center Cleveland Ohio USA

**Keywords:** actin‐binding proteins, breast cancer, cytoskeleton, TNBC, WAVE3

## Abstract

Breast cancer, a prevalent global health concern, has sparked extensive research efforts, particularly focusing on triple negative breast cancer (TNBC), a subtype lacking estrogen receptor (ER), progesterone receptor, and epidermal growth factor receptor. TNBC's aggressive nature and resistance to hormone‐based therapies heightens the risk of tumor progression and recurrence. Actin‐binding proteins, specifically WAVE3 from the Wiskott–Aldrich syndrome protein (WASP) family, have emerged as major drivers in understanding TNBC biology. This review delves into the intricate molecular makeup of TNBC, shedding light on actin's fundamental role in cellular processes. Actin, a structural element in the cytoskeleton, regulates various cellular pathways essential for homeostasis. Its dynamic nature enables functions such as cell migration, motility, intracellular transport, cell division, and signal transduction. Actin‐binding proteins, including WAVE3, play pivotal roles in these processes. WAVE3, a member of the WASP family, remains the focus of this review due to its potential involvement in TNBC progression. While actin‐binding proteins are studied for their roles in healthy cellular cycles, their significance in TNBC remains underexplored. This review aims to discuss WAVE3's impact on TNBC, exploring its molecular makeup, functions, and significance in tumor progression. The intricate structure of WAVE3, featuring elements like the verprolin–cofilin–acidic domain and regulatory elements, plays a crucial role in regulating actin dynamics. Dysregulation of WAVE3 in TNBC has been linked to enhanced cell migration, invasion, extracellular matrix remodeling, epithelial‐mesenchymal transition, tumor proliferation, and therapeutic resistance. Understanding the role of actin‐binding proteins in cancer biology has potential clinical implications, making them potential prognostic biomarkers and promising therapeutic targets. The review emphasizes the need for further research into actin‐binding proteins' clinical applications, diagnostic value, and therapeutic interventions. In conclusion, this comprehensive review explores the complex interplay between actin and actin‐binding proteins, with special emphasis on WAVE3, in the context of TNBC. By unraveling the molecular intricacies, structural characteristics, and functional significance, the review paves the way for future research directions, clinical applications, and potential therapeutic strategies in the challenging landscape of TNBC.

## 1 INTRODUCTION

Breast cancer is one of the most common diseases that affects a large number of women universally, thus significantly increasing women's health concerns (Sharma et al., 2010). Among the types of breast cancer, triple negative breast cancer (TNBC) is known to be more severe than others because it lacks three receptors: namely, estrogen receptor (ER), progesterone receptor (PR), and human epidermal growth factor receptor (HER2) (Augoff et al., 2012; Foulkes et al., 2010). Therefore, it is difficult to target this subtype through a hormone‐based therapy, leaving patients at a higher risk of tumor progression and recurrence. Moreover, it is an aggressive type of cancer that lacks proper prognosis due to the heterogeneity of its molecular makeup (Kumar & Aggarwal, 2016).

Currently, extensive research is underway to comprehend the molecular composition, genetic markers, epigenetics, and treatment modalities associated with this disease, all aimed at saving countless lives. Amidst these investigations, one pivotal variable must not be overlooked: actin‐binding proteins (Sun et al., 2008). Many researchers have diligently explored these proteins, elucidating their impact on the progression and metastasis of cancer cells, particularly in TNBC. Notably, these proteins possess the ability to construct and reconstruct the intracellular cytoskeleton, playing a crucial role in advancing TNBC tumors (Arumugam et al., 2021; Sossey‐Alaoui, 2020). Within the realm of actin‐binding proteins, our focus centers on a member of the Wiskott–Aldrich syndrome protein (WASP) family: WAVE3. Research indicates that WAVE3 could be a significant factor in the regulation of TNBC tumor biology (Frugtniet et al., 2015). This review seeks to unravel the intricate nature of TNBC progression and explores the specific role of WAVE3 in initiating, advancing, and metastasizing TNBC tumors (Davuluri et al., 2014; Kulkarni et al., 2012). After evaluating existing research data, the subsequent sections of this review will expound on actin‐binding proteins, delving into their structure, significance, and their contribution to cytoskeleton formation. A comprehensive discussion on WAVE3 and its significance in TNBC will follow. Finally, we will explore prognosis and treatment options for TNBC by targeting WAVE3, shedding light on a new aspect of breast cancer biology and therapeutics.

### 1.1 Roles of actin in physiological conditions: Fundamentals and function

Before we delve into the role of WAVE3 in the larger context of TNBC, a strong understanding of both functional and structural significance of actin, the protein that plays the role of one of the primary structural elements of the cellular cytoskeleton, is required (Kelpsch & Tootle, 2018). Actin is an important factor in coordinating a variety of cellular functions necessary for homeostasis and healthy cellular activity under normal physiological conditions (Sossey‐Alaoui et al., 2007; Wesolowska & Lénárt, 2015).

### 1.2 Actin is the structure of the cellular cytoskeleton

Human cytoskeleton is a complex assembly of structurally and functionally different proteins that are required for the maintenance of the cellular structure and its operation. Actin belongs to a group of proteins which are flexible in nature and consists of a globular structure (Tee et al., 2015). This globular structure is responsible for the formation of the microfilaments. It is also known as F‐actin, playing a significant part in cytoskeleton remodeling. The molecules of actin have the ability to polymerize as they can bind to each other and form long thread‐like microfilaments. The role of these microfilaments is to maintain the structural stability of the cells (Von der Ecken et al., 2015). They also serve as the scaffolding of the cells and thus help maintain their form and integrity. Moreover, the importance of actin extends beyond this role. The presence of microfilaments makes the process of polymerization and depolymerization easier due to their active nature. This also enables cells to respond quickly to their changing needs (Kadzik et al., 2020).

Not only do they play a major role in the formation and stability of actin cytoskeleton, but these small structural filaments are also important for the motility and movement of the cells. These microfilaments are responsible for pushing the membrane and creating space when the polymerization process starts occurring at the leading edge of the cell (Romero et al., 2020). This movement further helps in tissue formation through cellular migration. They also play a significant role in the process of cell division. The structure formed by these filaments contract at the time of cell division to separate the emergent two cells. This ability to polymerize as well as depolymerize is crucial for the cytokinesis process (Pollard & O'Shaughnessy, 2019). Actin also provides assistance with intracellular transportation. It coordinates with smaller molecules, including myosin, to facilitate the traffic of other components in and out of the cells. This is one of the most important mechanisms to ensure basic cell functioning (Henson et al., 2017).

### 1.3 Actin in cell migration and motility

This section discusses the detailed role of actin in various functions, including cell growth, migration, motility, and survival in physiological conditions. It is necessary for cells to organize themselves according to their changing needs and evolve based on their cellular environment (Svitkina, 2018). Therefore, to help them explore their environment, cells develop extensions at the cell surface known as lamellipodia or filopodia. This is one of the main processes that highlight the importance of actin in cellular movement (Leijnse et al., 2015). Lamellipodia or filopodia are important for cell motility during wound treatment or healing, immune responses, immune reactions, and significantly during embryo development. The structure of filopodia is slender with finger‐like projections at the outer part of the migrating cell. On the other hand, lamellipodia resemble sheet‐like projections located at the front of migrating cells. They contain an abundance of actin filaments controlled by a number of the actin‐binding proteins (Mueller et al., 2017).

The actin related proteins 2/3 (Arp2/3) complex and formins are also two important actin‐binding proteins that are crucial regulators of actin dynamics. Their function is to form and polymerize actin filaments. The polymerized actin filaments are further responsible for developing membrane protrusion (Molinie & Gautreau, 2018). These proteins work with each other and also help push the cell membrane further, which helps the cells move in the right direction. Cells detect changes in the environment or stimuli and respond by moving in that direction. Depending on the type of signal sensed, they might also move in a different direction from the stimulus. This sense of detection is crucial for the process of cell migration (Swaney & Li, 2016). Nevertheless, the actin cytoskeleton is also responsible for the formation of the contractile machinery, which is required for cell migration. In order to fight through adverse microenvironments, cells sometimes have to fit into very narrow spaces and alter their size and/or shape. To assist in this process, myosin, also known as a motor protein, along with actin, is responsible for producing the requisite contractile forces (Sweeney & Holzbaur, 2018). These forces allow the cells to change their form and fit into targeted spaces. This is important for immune cells as they need to react and move quickly to fight foreign agents and reach specific sites of injury or infection (Molinie & Gautreau, 2018).

Actin not only plays a role in the functioning of healthy cells under physiological conditions, but also plays a significant role in the development of deadly diseases such as cancer, which starts with cells exhibiting anomalous behaviors. These cells develop abnormal cellular movements, with excessive motility and invasion ability of healthy microenvironment (Suresh & Diaz, 2021). This invasive nature of the cancer cells also affects how actin dynamics work. This change can lead to uncontrolled cell migration to different tissues, causing metastasis (Sossey‐Alaoui et al., 2011). Therefore, it is important to understand actin dynamics and its interaction with other proteins in cell motility, both in physiological processes and in tumor progression (Izdebska et al., 2020).

### 1.4 Actin orchestrates intracellular transport

As discussed above, intracellular transportation is an important process facilitated by actin‐binding proteins. Actin also plays a role in the formation of various cellular components, which includes organelles and vesicles, and their proper placement within the cell. This entire process depends on transient interactions between these microfilaments and myosin (Jung et al., 2019). The microfilaments take the lead for the myosins to follow. These motor proteins play a special role in utilizing adenosine triphosphate as their energy source for movement. This process allows the transport of cargo, including required chemicals, small molecules, and large organelles. Mitochondria and endoplasmic reticulum (ER) are among the transported organelles. The ER is responsible for protein synthesis and the metabolism of different lipid compounds, requiring their transport to specific locations for their proper cellular function (Kast & Dominguez, 2017). **Actin** proteins help in this process and ensure that these organelles are placed at the correct locations. There are different types of transport processes that occur with the help of actin‐binding proteins. Among them, axonal transport takes places in neurons and is a type of intracellular transport (Wu et al., 2017). Microtubules, another element of cytoskeletal components, also play a significant role in the transport of neurotransmitters within axons. This process is important for the proper functioning of the nervous system and synaptic activity. Thus, actin is a crucial element for regulating all aspects of intracellular transport processes in live cells (Kapitein & Hoogenraad, 2015).

### 1.5 Cell division

Cell division is the primary and the most important process in all living entities. To ensure this process occurs in a regulated manner, the dynamics of actin needs to be understood. Cytokinesis is the process in which actin is involved to ensure that cell division occurs smoothly to result in the exact replication of the genetic material. This is important for the growth and development of the corresponding daughter cells (Rottner et al., 2017). Cytokinesis takes place at the end of the entire cell division process, once the separation of the chromosomes occurs through mitosis and meiosis phases. In this process, actin ensures the physical division of the mother cells into daughter cells, which are genetically alike (Dogterom & Koenderink, 2019). Actin creates a ring known as the contractile ring and regulates it during the initial phase of cytokinesis through the process of polymerization on the surface of the cells. Motor proteins, including myosins, coordinate with the actin filaments, which then provide a contractile force for the division of the cells (Schwayer et al., 2016). Later, the contractile ring formed by actin divides the mother's cytoplasm and organelles into two. Thus, this process is very critical and requires actin and any malfunction could result in fatal consequences (Kruppa & Buss, 2021).

### 1.6 Signal transduction

The role of actin filaments in the process of signal transduction is also well known to researchers. The filaments are responsible for enabling signal molecules to interact with extracellular signals, thereby allowing them to enter the nucleus. This entire process is known as signal transduction, and it is important for regulating various cellular processes including proliferation, differentiation, and gene expression (Humphries et al., 2019). Thus, actin multitasking between its various roles engages many vital functional aspects under physiological conditions. This regulated process is important for maintaining a healthy cellular environment. Many actin‐binding proteins regulate its dynamic features, enabling cells to adapt to changing conditions and carry out vital life‐sustaining processes. Therefore, understanding the influence of actin on adverse conditions such as TNBC is necessary and may pave the way not only for unraveling key mechanisms of the development of this disease but also for discovering new potential therapeutic modalities (Rigiracciolo et al., 2020).

### 1.7 Actin‐binding proteins‐ function in the proliferation of tumors

The interaction of actin‐binding proteins and their role in the structure of cytoskeleton also has a significant impact on the growth of the tumor cells, their proliferation, migration, and ultimately metastasis. They also play a significant role in modulating the dynamics of the cytoskeletal structure (Izdebska et al., 2018). This whole process affects various cellular cycles, which results in tumor formation.

#### 1.7.1 Actin binding proteins and their mechano‐sensitive regulation

Evolving studies demonstrate that actin‐binding proteins are controlled by mechanical signals from the cancer microenvironment (Chaqour & Goppelt‐Struebe, 2006). These signals used by nearby cells and the extracellular matrix (ECM) may impact the movement and localization of these proteins, affecting cell propagation and their movement (Eroshkin & Zaraisky, 2017). For instance, the protein called zyxin plays a supporting role for actin‐binding proteins at primary linkages and is responsible in interpreting the signals and the modification in actin dynamics (Ermolina et al., 2010).

#### 1.7.2 Actin‐myosin contractility in cancer progression

These proteins do not only maintain the dynamics of actin but they also play a role in the activation of myosin motors for the generation of contractile forces inside the cells (Unbekandt & Olson, 2014). Current research highlights the significance of actin‐myosin contractility in influencing cancer cell proliferation by supporting cellular shape, deformation process of the nucleus, and the segregation of chromosomes during the cellular process of mitosis (Gibieža & Petrikaitė, 2021). Aiming for more studies related to the actomyosin cytoskeleton could demonstrate a novel strategy in the prevention of the cancer progression.

#### 1.7.3 Posttranslational modification

Actin‐binding proteins pass through several posttranslational modifications (PTMs) stages which are responsible for maintaining their active roles in the progression of cancer (Terman & Kashina, 2013). For example, actin binding proteins, namely, cofilin and cortactin are phosphorylated by kinases such as LIM kinase and Src kinase, a PTM event that alters their binding affinity for actin filaments and modulates division of cells and their invasive property (Shishkin et al., 2016). Considering the regulatory function of these modifications in actin‐binding protein function might disclose novel remedial possibilities for cancer management.

#### 1.7.4 MicroRNA regulation

MicroRNAs also called miRNAs are tiny non‐coding RNAs that play an important role in the regulation of gene expression by targeting mRNAs once the process of transcription is completed. Current reports have discovered miRNAs that target actin‐binding proteins and control their expression in tumors (Uray et al., 2020). For instance, the miR‐200 family plays a role in the suppression of expression of fascin, an actin‐binding protein, and as a result, inhibits cancer cell migration (Fu et al., 2020). Controlling the regulatory property of miRNAs on actin‐binding proteins could provide novel strategies for cancer treatment.

#### 1.7.5 Cellular inactivity

Although most of the research efforts have focused on the role of actin‐binding proteins in the proliferation of cancer tumors, current data demonstrates that these actin‐binding proteins play additional crucial roles in regulating cellular quiescence (Lee & Dominguez, 2010). Drebrin and tropomyosin are examples of such proteins, since they maintain the association of actin filaments in quiescent cells, affecting their sensitivity to their surroundings as well as to the growth signals (Middelbeek et al., 2016). Considering how these proteins play a dual role in the transition that occurs between the proliferative and quiescence stage could provide more insights on why and how they recur.

#### 1.7.6 Extracellular functions

In addition to their intracellular functions, these proteins are also found to exert their effects at the extracellular level to regulate cancer progression and metastasis (Mannherz & Hannappel, 2009). For instance, gelsolin can be released by tumor cells and stimulate ECM remodeling, therefore, activating cancer cells proliferation (Feldt et al., 2018). Further investigation on the extracellular roles of these proteins might reveal new processes of tumor interactions and identify novel targets for cancer therapy.

## 2 THE RHO‐ROCK PATHWAY, ARP2/3 COMPLEX AND WASP/WAVE PROTEIN FAMILY, AND THE IMPORTANT ROLES THEY PLAY IN MODULATING TUMOR GROWTH

### 2.1 Rho‐ROCK signaling and its involvement in cancer progression

**FIGURE 1:**
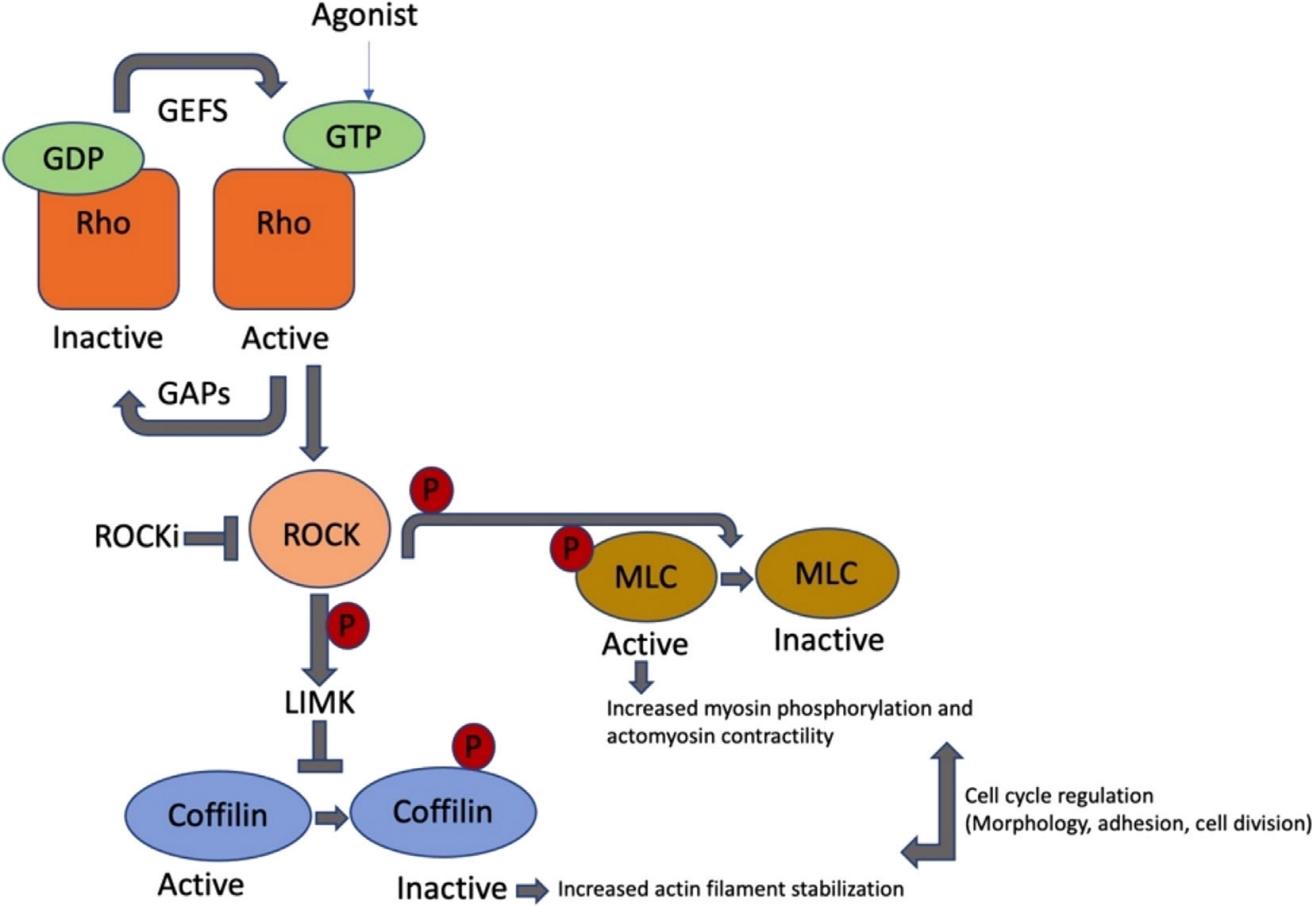
Rho‐ROCK signaling pathway in cell cycle regulation (Chin et al., 2015).

The Rho‐ROCK pathway is a complex signaling cascade involving the Rho family of GTPases and the Rho‐associated protein kinase (ROCK). It regulates the intricacies of actin dynamics with other proteins, and has an important impact on many cellular functions (Afzal & Sadaf, 2019). This pathway is significantly important for a wide range of biological functions, including the determination of cell types, their migratory mechanisms, and tissue formation. It derives its name from the expression of the ROCK (Narumiya & Thumkeo, 2018). This protein is responsible for maintaining and regulating the activity of the important proteins, which are responsible for the development and the regulation of the actin cytoskeleton (Figure 1). This pathway is triggered by the enzymatic activation of Rho GTPase or RhoA (Wu et al., 2023). These are small molecules that act as molecular switches responsible for converting an inactivated GDP‐bound form to an activated GTP‐bound state. The phosphorylation and activation of ROCK is mediated by the enzymatic activity of RhoA. Phosphorylated ROCK then affects actin cytoskeleton through different mechanisms. ROCK primarily targets the myosin light chain (MLC). This causes the change in the shape of myosin, resulting in increased actomyosin contractility, which is important for cellular processes (Johan & Samuel, 2019). Thus, MLC phosphorylation is responsible for the contractility in actomyosin, which accounts for other cellular processes (Figure 1). Cells also use their membrane extensions and actin‐based contractile pressure to their advantage. They use this pressure to propel and migrate from one place to another. This pathway also provides integrity to the tissues by producing cellular junctions and required cell‐matrix adhesions (Valencia‐Expósito et al., 2016). In short, Rho‐ROCK system is crucial for regulating and maintaining cell shape and size by producing actin filaments as well as focal adhesions. This process is also a key factor in the development of various tissues and organs. A dysfunction in the Rho‐ROCK pathway results in the development of cancer (Narumiya & Thumkeo, 2018).

Dysregulated Rho‐ROCK pathway is believed to be connected to the promotion of tumor growth by enhancing cellular contractility as well as promoting mitotic processes. Efficient separation of cells is encouraged because cytokinesis is assisted by enhanced contractility. Rho‐ROCK signaling also has the potential to regulate gene expression, which affects cell cycle and cell proliferation (Wei et al., 2016).

### 2.2 The Arp2/3 complex

The Arp2/3 complex plays an important role in the complicated world of actin cytoskeleton dynamics. This complex is essential for the regulation of actin branching and nucleation, both of which are required for the creation of multilayered structures in cells and for motility (Pizarro‐Cerdá et al., 2017). Actin filaments are normally long linear polymers, although the creation of branched networks is essential for a variety of biological processes. This sophisticated process is orchestrated by the Arp2/3 complex. It is composed of seven distinct subunits and is appropriately named, since it includes two actin‐related protein molecules, Arp2 and Arp3, as well as other subunits (Swaney & Li, 2016).

The Arp2/3 complex, as one of its main activities, catalyzes the formation of newly synthesized filaments from the sides of existing actin parent filaments. The Arp2/3 complex binds to the existing filament of actin and initiates the formation of “daughter” filaments at a unique angle. A dendritic actin system is produced when many offspring filaments break out from a single parent filament. This actin branching is critical for many cellular structures and activities (Swaney & Li, 2016). Lamellipodia and filopodia, specialized membranes extensions at the leading edge of migratory cells, rely largely on the Arp2/3 complex. Filopodia have slender, and finger‐like protrusions that sense the surroundings and help steer directional movement, whereas lamellipodia are expansive, sheet‐like extensions that facilitate cell movement. Furthermore, the Arp2/3 complex is essential for the creation of additional actin‐rich frameworks, such as branch actin pathways at the cell periphery and the dendritic actin meshwork observed underneath the plasma membrane (Gallop, 2020). These structures are essential for preserving cell shape, enabling intracellular communication, and supporting numerous cell functions. The Arp2/3 complex is carefully regulated. Signaling pathways triggered by cell surface receptors, along with numerous actin‐binding proteins, play critical roles in controlling the structure and function of the Arp2/3 complex. Such biological processes mean that actin branching happens in a geographically and temporally controlled manner, helping cells to quickly adapt to their constantly shifting environments and accomplish complicated tasks such as cell migration, tissue formation, and immunological responses (Molinie & Gautreau, 2018).

### 2.3 Effect on the proliferation of tumors

One of the distinguishing features of tumor cells is increased cell‐to‐cell movement and intrusiveness, which can be produced by aberrant actin polymerization mediated by Arp2/3 complex dysregulation. Actin‐rich extensions can help tumor cells spread by enhancing their motility. The Arp2/3 complex also regulates cell adherence and ECM modification, both of which are required during tumor cell invasion into surrounding tissues (Shortrede et al., 2016).

### 2.4 WASP/WAVE family of proteins

The family of WASP/WAVE (WASP‐family Verprolin‐homologous Protein) is an interesting protein family with a common domain structure that comprises important complexes and factors, including actin nucleation‐promoting factors. This family of proteins is also present and plays a role in the control of complex cellular cytoskeleton. These proteins play a major role in activating the Arp2/3 system and thereby catalyzing the nucleation and branching process of actin filaments (Frugtniet et al., 2015). They have significant effects on the actin polymerization process as well as cellular activities. They also help in the formation of specialized structures such as lamellipodia and filopodia. The principal functional goal of this family is to activate the Arp2/3 complex to promote actin branching. The resulting filament network formed is essential for different cellular activities. An example of this is lamellipodia, which are broad, sheet‐like structures containing extensions that are crucial for cell movement (Rottner et al., 2021). They help cells migrate from one location to another by extending their leading edge. This process is highly dependent on the rate of nucleation and the division of the actin filaments, which are controlled by this family of proteins (Kage et al., 2022).

Another example like lamellipodia is filopodia. Contrary to lamellipodia, filopodia are slender and weaker. They contain finger‐like protrusions that play a significant role in sensory processes as well as motility. The formation of these structures also relies on actin dynamics, which are controlled by this protein family as they play a role in other developmental processes. The regulation of activity of this family is complex (Pipathsouk et al., 2019). They are sensitive to different cellular processes and pathways, which could result in their activation or inhibition. This sensitivity could be triggered through stimuli or receptors on the surface of the cells. Examples such as RhoA, Cdc42, and Rac1are regulatory members of this family. Once these members are activated, they result in an activation cascade which culminates in the stimulation of the Arp2/3 complex. Additionally, this family plays an essential role in cell adhesion, neuronal development, and immunological responses. Any disruption in this process can lead to immune disorders and give rise to various cancers (Whitelaw et al., 2020).

### 2.5 Regulatory mechanisms of WASP/WAVE proteins

These proteins play a primary role as key regulators of cytoskeleton remodeling. They accomplish this by networking with numerous regulatory compounds and multiprotein centers, including Arp2/3 complex, to facilitate the association of actin filaments (Takenawa & Miki, 2001). This mechanism, recognized as actin nucleation, is important for producing the stimulus essential for protrusions, dynamics of the membrane, and intracellular trafficking (Takenawa & Suetsugu, 2007). Moreover, these proteins show necessary functions in organizing actin polymerization with cellular mechanisms, including endocytosis as well as cytokinesis (Pollitt & Insall, 2009).

The engagement of proteins in cytoskeleton remodeling is closely controlled by an intricate connection of regulatory processes that manage their spatial and temporal dynamics inside the cell (Padrick et al., 2008). One important regulatory process includes the attachment of regulatory compounds, which includes Rho GTPases and phosphoinositides, to certain domains in these proteins. These connections modulate the configuration of the proteins, leading them to react vigorously to extracellular as well as intracellular cues (Padrick & Rosen, 2010). Additionally, PTMs, such as the processes of phosphorylation, acetylation, and ubiquitination are responsible for regulating the stability, and localization of these proteins (Takenawa & Miki, 2001).

They serve as signaling hubs, incorporating extracellular cues with intracellular remodeling to align cellular reactions to surrounding signals. Throughout their connections with numerous signaling compounds and proteins, these proteins modulate different pathways involved in cell propagation, and division (Alekhina et al., 2017). Moreover, dysregulation of these proteins is shown to be associated with the pathology of several diseases, various cancers, and neurological disorders, therefore, underlining their significance in medical conditions (Rottner et al., 2021).

### 2.6 Role WASP/WAVE proteins in cancer progression

Dysregulation of WASP/WAVE proteins could lead to malignant progression of cancer cells, and thus, deciphering the structure/function of this family of proteins may be a significant step toward understanding their function in the regulation of cytoskeletal dynamics and in cancer biology. The dysregulation of this protein family is responsible for promoting cellular activities such as proliferation, invasion and activation of signaling pathways that support cell survival (Frugtniet et al., 2015). The literature provides ample evidence linking dysregulation of activity of the WASP/WAVE proteins to the aggressiveness of various types of cancers, through enhanced tumor cells proliferation, migration and invasion. This irregular process is important in the invasive nature of tumors, leading to metastasis (Kramer et al., 2022). Dysregulated WASP/WAVE proteins, in addition to promoting cancer growth by increasing cell motility and migration, also help in the formation, organization, or reorganization of actin filaments and the development of lamellipodia or filopodia. This helps cancer cells in migrating through various tissues and structures. In addition, this family is identified to work in collaboration with different signaling pathways that are not functioning properly in cancer (Zheng et al., 2023).

### 2.7 WASP/WAVE family proteins' structural characteristics

This family can be distinguished due to its diverse structural topography, which plays an important role in controlling the actin cytoskeleton (Tyler et al., 2016). The structural characteristics are described in detail below.

#### 2.7.1 The verprolin–cofilin–acidic domain

This domain is located at the C‐terminus of the WAVE/WASP proteins and plays a significant role in enabling these proteins to regulate actin dynamics. This domain is composed of three different regions, namely: verprolin (V), cofilin (C), and acidic (A). These subregions are internally connected to each other and help in the nucleation and branching of actin (Frugtniet et al., 2015). **(i)** The **Verprolin (V)** is responsible for helping different monomers bind to each other such as (G‐actin) or filamentous (F‐actin) actin. It also stimulates the nucleation process through the attraction between actin monomers, inducing the formation of filaments (Fernando et al., 2008; Toms, 2016). **(ii)** The **Cofilin (C)** domain is responsible for binding to actin filaments, causing depolymerization. It also promotes the formation of actin filament ends, which are essential for the nucleation through Arp2/3 complex (Mughees et al., 2021). **(iii)** The **Acidic (A)** region is responsible for the interaction with the Arp2/3 complex. This connection is critical for the activation of the Arp2/3 complex and the initiation of branching of actin networks (Tyler et al., 2016).

#### 2.7.2 Interaction with signal transduction molecules

The N‐terminal region present on these proteins enables them to bind to different signaling molecules, including Rho GTPases, which are important regulators of actin dynamics. These connections allow extracellular cues to activate or inhibit the WASP/WAVE family members (Frugtniet et al., 2015).

#### 2.7.3 Regulatory domains

Some members of the family may have extra regulatory domains located in the middle of the protein, between the N‐terminus region and the VCA domain. These regulatory domains can mediate responses to various factors, such as different actin‐binding proteins or scaffold proteins, which can further modulate their activity and selectivity.

The inclusion of the VCA domain in each member from the WASP/WAVE family appears to be a distinguishing structural trait that emphasizes their shared involvement in stimulating actin polymerization and branching. On the other hand, the variability in their N‐terminal sections provides for different levels of specialization and control, allowing these proteins to participate in cellular processes other than actin cytoskeleton regulation (Rana et al., 2021; Tyler et al., 2016).

### 2.8 The WAVE3 protein

**FIGURE 2:**
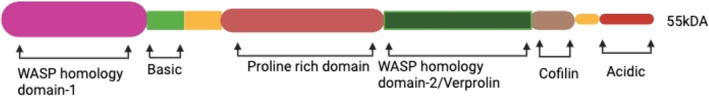
The representation of the structural domain of WAVE3 (Kollmar et al., 2012).

WAVE3 belongs to the WASP family. It acts as a key regulator of actin cytoskeleton, applying intense stimulus over various cellular mechanisms. In this family, WAVE3 can be differentiated by its distinctive structure and complex functions, demonstrating itself as a key player in the organization of the cellular dynamics. Structurally, while it share the core domain structure with other members of the WASP/WAVE family (Figure 2), it also contains specific variations, helping its complex functionality in cellular settings (Kansakar et al., 2020).

WAVE3 represents an integrated structure depicted by several operative domains that assist its different cellular roles (Figure 2). The N‐terminal region of WAVE3 is responsible for the protection of the WH1 (WASP homology 1) domain, necessary for connections with other proteins in the WAVE regulatory complex (WRC) and for controlling the process of actin polymerization. Bordering the WH1 domain is the proline‐rich region, aiding as an attachment location for proteins in signaling mechanisms and the regulatory process of cytoskeleton (Sossey‐Alaoui et al., 2005). The C‐terminal region of WAVE3 involves the verprolin homology (V) domain, which is responsible facilitating binding to the actin‐related protein 2/3 (Arp2/3) complex, thus supporting actin nucleation processes along with filament branching (Mizutani et al., 2005).

The function of WAVE3 is entangled with its role as a primary facilitator of actin cytoskeleton. WAVE3 joins with other subunits to stimulate the assemblage of actin filaments, aiding cellular mechanisms including cell movements, membrane protrusion, and the process of endocytosis. The supportive action of WAVE3 and the Arp2/3 complex helps the creation of actin networks, crucial for producing the force essential for cellular movements (Takenawa & Suetsugu, 2007). Its functional range outspreads to the maintenance of intracellular signaling pathways as well as cellular reactions to extracellular signals. By its connections with several signaling compounds and cytoskeletal effectors, it impacts a wide range of cellular mechanisms, comprising cell attachment, cell cycle proliferation, and cell–cell interactions (Takenawa & Miki, 2001). Additionally, dysregulation of its expression is associated with pathological conditions, including tumor metastasis, and autoimmune disorders, highlighting its importance as a potential therapeutic target (Sossey‐Alaoui et al., 2007). Current reports have discovered a unique association between expression of WAVE3 and the immune evasion process in different types of cancer (Lee et al., 2021). Although conventionally reviewed for its function in the regulation of actin cytoskeleton dynamics and tumor cell invasion, WAVE3 might affect the cancer immune microenvironment (Kulkarni et al., 2012). The following points discuss the WAVE3 role in antitumor immune evasion (Kulkarni et al., 2012).

### 2.9 WAVE3 mediates the host immune suppression

Evolving data proposes that the expression of WAVE3 in tumor cells might support immune evasion through the modulation of the immune expression of checkpoint molecules and inhibitory receptors. Increased WAVE3 levels are shown to be linked with high expression of programmed death‐ligand 1 which is responsible for the inhibition of anticancer immune reactions by hindering T cell initiation and stimulating T cell collapse (Wanka et al., 2020). This process of WAVE3 could possibly favor an immunosuppressive cancer microenvironment, that is resistant to immunotherapies.

#### 2.9.1 Tumor infiltrating lymphocytes

The expression of WAVE3 in tumors could impact the configuration and role of tumor infiltrating lymphocyte (TILs), that play a crucial function in antitumor immune response. Research showed increased WAVE3 expression associates with decreased infiltration of cytotoxic T lymphocytes. In addition, natural killer cells are also reduced into the cancer microenvironment, damaging cancer cell death and immune surveillance (Chen et al., 2017).

#### 2.9.2 Predictive biomarker

WAVE3 expression in cancer tissues may help as a prognostic biomarker for patients' response to immunotherapies. Patients with tumors that have increased WAVE3 expression might display decreased reaction to immune checkpoint inhibitors (ICIs) because of its immunosuppressive property. Consequently, WAVE3 expression levels may serve in classifying patients who are doubtful to benefit from ICIs and could possibly need an alternative/combination therapy (Wanka et al., 2020).

#### 2.9.3 Targeting WAVE3

Targeting WAVE3 in combination with immune checkpoint blockade signifies a hopeful therapeutic approach to improve antitumor immune reactions and treatment outcome. Preclinical findings have determined that WAVE3 knockdown sensitizes cancer cells to the immune‐mediated killing and boosts the efficiency of immunotherapy in in vivo animal models. Combination of WAVE3 inhibitors with other immunomodulatory means might overcome the resistance process and synergistically prevent tumor relapse (Moon et al., 2021).

#### 2.9.4 Clinical translation

More in depth clinical studies are required to decipher the detailed process for the WAVE3‐mediated immune modulation in tumors and to confirm its function as a prognostic biomarker in clinical settings. Research assessing the efficiency of WAVE3 inhibitors alone or in combination with other therapies, are necessary to consider regarding their safe use with patients.

### 2.10 WAVE 3 compared to other WAVE isoforms

WAVE3 regulation includes a multifaceted interaction of numerous signaling compounds, PTMs, and various expression arrays in tissues. WAVE isoforms share structural domains and essential regulatory processes. Several alterations exist in the regulatory mechanism of WAVE3 that point to its novel role and cellular functions (Kansakar et al., 2021). They display diverse expression patterns that are specific to the tissues associated with other isoforms. While WAVE1 and WAVE2 isoforms are universally present in several cell types and tissues, WAVE3 expression is mainly seen in neural tissues, including the brain and spinal cord (Tang et al., 2020). The spatial and temporal differential expression profile between the different WAVE isoforms proposes specific roles for each isoform. WAVE3 connects with supervisory compounds and signaling pathways in an approach different from other WAVE isoforms, affecting its movement and subcellular localization (Nozumi et al., 2003). For instance, WAVE3 has been demonstrated to relate with particular guanine nucleotide exchange factors and GTPase‐activating proteins regulating the action of Rho GTPases, such as Rac1 and Cdc42. This ability stimulates actin nucleation and cytoskeletal remodeling (Nozumi et al., 2003).

### 2.11 WAVE 3 role in other human diseases

Although lot of the data on WAVE3 focuses on its contribution in cancer, mainly in triple‐negative breast cancer (TNBC), research proposes its role in many other human diseases.

#### 2.11.1 Neuronal development

Its expression is found during the developing stages of the nervous system and is associated with several processes including neuronal relocation, axon control, and dendritic spine morphogenesis (Ponimaskin et al., 2007). Data from in‐vivo animal models demonstrate that its deficiency or reduced expression can result in abnormalities including autism spectrum disorders as well as intellectual disabilities (Muñoz‐Lasso et al., 2020).

#### 2.11.2 Synaptic plasticity

WAVE3 is also linked to the regulation of synaptic plasticity, an important mechanism required for learning and memory. By controlling actin cytoskeletal dynamics at the synapse, it might impact synaptic structure and function which could cause diseases such as Alzheimer's and schizophrenia (Peebles, 2009).

#### 2.11.3 Activation and migration of immune cells

WAVE3 has an important function in the migratory process of immune cells, mainly trafficking the leukocytes and causing inflammation. It controls cytoskeletal dynamics in these cells, promoting their migration toward the infection sites (Park & Kim, 2017).

#### 2.11.4 T cell function

WAVE3 is also involved in controlling activation of T cells and their effector roles. It impacts the production of synapses between these cells and antigen‐presenting cells. Additionally, it also regulates dynamics of signaling complexes with T cell receptors. Dysregulation results in autoimmune disorders and immunodeficiency syndromes (Huang & Burkhardt, 2007).

#### 2.11.5 Vascular remodeling

WAVE3 is expressed during the migration of vascular smooth muscle cells (VSMCs), a process that is crucial for vascular remodeling, as well as repairing and healing after an injury (Uray et al., 2020). Dysregulated expression or function in VSMCs could lead to several pathological conditions, for example, atherosclerosis, and hypertension (Uray et al., 2020).

Hence, WAVE3 is evolving as a complex regulator of different mechanisms and cellular functions outside the context of cancer. Its contribution in these deadly diseases underlines its importance in various physiological processes. More research on its cellular and molecular mechanisms may help in discovery of novel therapeutic targets to treat these diseases.

### 2.12 Dysregulation of WAVE3 and other WAVE isoforms in different cancers

WAVE3 has been shown to contribute the pathology of different types of cancers. In colorectal cancer, for example, WAVE3 contribution to cancer proliferation is associated with metastasis. Research has discovered complicated mechanisms through which WAVE3 plays a role in tumor aggressiveness, by controlling cell adhesion, relocation, as well as epithelial‐to‐mesenchymal transition (EMT) (Wang et al., 2020). By modulating the mesenchymal‐like phenotypes, the tumor cell motility and invasive phenotypes are improved, therefore, enabling metastasis. Furthermore, dysregulated expression of WAVE3 is linked with progressive cancer stage, and poor prognosis. Likewise, other cancer types including pancreatic cancer, skin cancer like melanoma, and tumors of glioblastoma are linked with both WAVE1 and WAVE2 isoforms, as they play a significant role in the aggressive nature of these types of cancers. This usually occurs due to their impact on cell migration, invasiveness, eventually leading to metastasis (Kunapuli et al., 2010; Rana et al., 2021; Wang et al., 2020). Specifically, pancreatic cancer is associated with dysregulated WAVE1 as it has a positive role in helping cancer cell invasion. This process occurs by forming the invadopodium, and other cellular structures that play major roles in the degradation of ECM during the invasion (Rana et al., 2021). Contrary to this, WAVE2 has been found to contribute to glioblastoma, by supporting invasion and the cancer cells proliferation as it regulates cell motility and actin cytoskeletal dynamics (Kunapuli et al., 2010). Moreover, in melanoma, both isoforms are associated with the invasive phenotype of these tumors, as they expression levels correlate with the aggressive nature of these tumors, leading to metastasis (Wang et al., 2020). These data underline the various roles of different WAVE isoforms in regulating tumor progression in different cancer types. By explaining the detailed mechanisms of WAVE family dysregulation in these aggressive tumors, scientists intend to discover novel cancer therapeutic targets to improve patient treatment.

In the setting of dysregulated gene expression in TNBC, researchers frequently emphasize understanding modifications in several genes and pathways that contribute to the aggressive nature of this subtype. Dysregulation in gene expression can result in irregular cellular processes, unrestrained proliferation, and resistance to conventional therapeutic methods. Examining the precise genes involved and their influence on TNBC pathogenesis is crucial for developing targeted treatments and improving treatment outcomes for patients affected by this aggressive type of breast cancer (Tata et al., 2019).

### 2.13 WAVE3 in TNBC metastasis and proliferation

**FIGURE 3:**
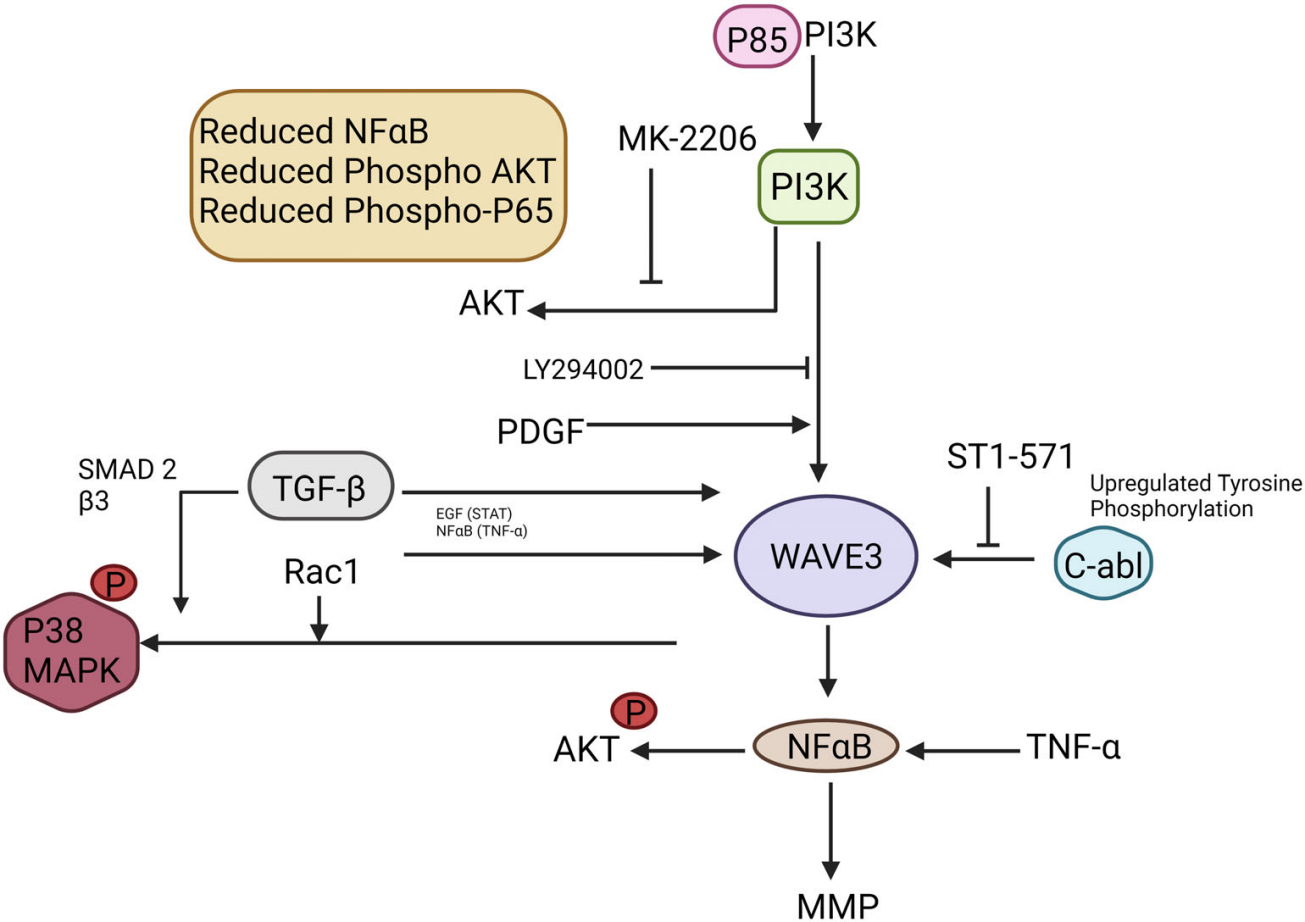
Summary of the molecular pathways where WAVE3 plays a significant role in the progression, invasion, and metastasis of cancer (Kansakar et al., 2020).

When compared with other subtypes of breast cancer, TNBC is even more malignant and is associated with increased risk of metastasis (Wang et al., 2023). WAVE3, also referred to WASF3, plays an essential role in the invasion‐metastasis cascade of TNBC tumors (Sossey‐Alaoui, 2013). WAVE3 has been implicated in several essential pathways linked with metastasis in TNBC including (Figure 3):

#### 2.13.1 Cell migration and invasion

WAVE3 has been found to enhance the migratory and invasive aspects of TNBC cells. It supports the formation of actin‐based assemblies in cells such as lamellipodia and filopodia, which are crucial for cell movement. WAVE3 promotes the migration of TNBC cells into the adjacent tissues by modifying the actin cytoskeleton, a fundamental phase in the metastatic cascade (Wang et al., 2020).

#### 2.13.2 ECM remodeling

Cancer cells must breach the ECM, a complex assembly of proteins that shield and support tissues, during metastasis. WAVE3 aids in transforming the ECM by modulating actin dynamics, allowing TNBC cells to navigate through the ECM and form secondary tumors in distance sites (Wang et al., 2020).

#### 2.13.3 Epithelial‐mesenchymal transition

EMT is a biological process that confers mesenchymal features to cancer cells, such as improved motility and invasiveness. WAVE3 has been associated with EMT stimulation in TNBC cells, facilitating a transition from a stable epithelial state to a migratory, mesenchymal phenotype. This critical transition is a major driver of metastasis (Kansakar et al., 2020).

#### 2.13.4 Tumor proliferation

WAVE3 could influence TNBC cell proliferation in addition to its role in metastasis as described above. It may contribute to tumor mass expansion at the primary site by shifting actin dynamics and possibly engaging other signaling pathways that are responsible for cell survival and proliferation (Kansakar et al., 2021).

#### 2.13.5 Therapeutic resistance

Numerous studies have associated WAVE3 overexpression in TNBC with the resistance to certain cancer therapies. This is particularly concerning because treatment resistance significantly reduces the effectiveness of chemotherapy and other targeted therapies in TNBC patients (Wang et al., 2023).

Because of its essential role in TNBC proliferation and metastasis, WAVE3 has emerged as a promising therapeutic target. Because WAVE3 is an active protein that interacts with actin and a number of other actin‐binding proteins, strategies that obstruct its activity or interactions may be valuable in slowing down the progression of this invasive breast cancer subtype (Neophytou et al., 2018). Understanding WAVE3's complex role as an actin‐binding protein is crucial for shedding light on the mechanisms that fuel TNBC invasion and for advancing new efficient treatment options. In the next section of this review, we will address the molecular processes by which WAVE3 plays a role in TNBC disease progression, how it interacts with other proteins, and the probability of using this information as a basis for innovative therapeutic techniques.

### 2.14 Clinical implications and biomarker possibilities

WAVE3 has significant clinical implications in TNBC. The association of elevated WAVE3 expression with unfavorable clinical outcomes has highlighted WAVE3's ability as a prognostic biomarker for TNBC. Additionally, understanding WAVE3's role in TNBC may help in the development of therapeutics (Bledzka et al., 2017). Methods that inhibit WAVE3 from functioning and interacting with a various actin‐binding partners might slowdown or impede TNBC progression. WAVE3's expanded role in TNBC may unlock the door to new therapeutic approaches. One actin‐binding protein that could possibly inhibit the growth and spread of TNBC is WAVE3. However, additional research exploration and development are required to achieve the efficient targeting of this marker for the purpose of halting the disease progression while maintaining its normal function that supports healthy cell function (Wang et al., 2023).

## 3 DISCUSSION

As already described, TNBC is a difficult as well as aggressive type of breast cancer that lacks expression of a number of receptors, including ER, PR, and human HER2 (Foulkes et al., 2010). This review has focused on the investigation of the expression of WAVE3 as one of the actin‐binding proteins in TNBC. This discussion will highlight significant details in terms of the consequences of the function of this protein and targeted pathways in TNBC research. WAVE3 is found to have elevated expression in TNBC compared to all other subtypes of breast cancer. Numerous pieces of evidence presented in multiple studies indicate reduced expression of WAVE3 in healthy cells. This differential expression of WAVE3 highlights its potential diagnostic value in TNBC. TNBC could be diagnosed earlier based on the expression of WAVE3 gene, potentially reducing mortality from this aggressive subtype. Further studies are required to understand WAVE3 expression and its clinical value in the development of standardized techniques to reliably determine the WAVE3 levels (Kansakar et al., 2021).

An essential factor in the context of TNBC is the significant role of WAVE3 in controlling actin cytoskeleton dynamics. Actin remodeling is necessary for numerous cellular functions, such as cell motility, invasion, and cell–cell interactions. Disordered actin dynamics contribute to the development of tumor cells motility and ultimately metastasis, which is a trait of TNBC's aggressiveness. Additional research is needed to fully understand the intricate mechanisms by which WAVE3 controls these cellular pathways. The potential effectiveness of WAVE3 as a targeted treatment modality in TNBC is one of the most exciting aspects of this research (Kansakar et al., 2021). Small interfering RNA or compounds targeting WAVE3 have shown promising effects in reducing TNBC cell migration and invasion in preclinical examinations. TNBC progression could be slowed with targeted therapeutics that alter the WAVE3‐mediated cytoskeleton actin dynamics. However, challenges such as efficient drug delivery and potential side effects must be taken into consideration during clinical therapy development (Chen et al., 2021).

### 3.1 Future directions

The literature study conducted during this review highlighted new directions implicating WAVE3 as a promising target for the treatment of patients with TNBC tumors, and probably with other tumor types. To better understand WAVE3, it is necessary to study the multiple signaling pathways involving WAVE3. It is also important to study the interaction of WAVE3 with its partner molecules in TNBC. Furthermore, the investigation of EMT could potentially lead to new therapeutic discoveries. The number of clinical trials in TNBC treatment needs to be significantly increased to help determine the prognosis and diagnosis value of WAVE3 in TNBC patients. Therapies in conjunction with other drugs targeting WAVE3 in TNBC patients also need to be tested (Bai et al., 2021).

## 4 CONCLUSION

In closing, this review has emphasized the significance of WAVE3 as one of the actin‐binding proteins of particular importance in the development and progression of TNBC. The expression of WAVE3 in TNBC, its role in cytoskeleton dynamics, and the potential of WAVE3 as a target in TNBC treatment have been presented. The evidence from the literature reviewed sheds light on the potential roles actin‐binding proteins play in the regulation of tumor pathology, particularly in TNBC, offering hope for both early diagnosis and treatment of this devastating disease. The mechanisms that underly the aggressiveness of this disease are probably the same that drive its poor prognosis. The continued investigation into the yet untapped role of WAVE3 in TNBC holds the potential and possibly the promise to make significant advances in uncovering these mechanisms.

## CONFLICT OF INTEREST STATEMENT

The authors declare no conflict of interest.

## Data Availability

Data sharing is not applicable to this article as no datasets were generated or analyzed during the current study.
